# Naringenin Inhibits Colorectal Cancer associated with a High-Fat Diet through Modulation of Gut Microbiota and IL-6/STAT3 Pathway

**DOI:** 10.4014/jmb.2412.12029

**Published:** 2025-04-23

**Authors:** Jiahui Sun, Luyao Shi, Fan Xu, Hanyan Sun, Yitong Liu, Jiangyun Sun, Qingxin Zhou

**Affiliations:** 1 Department of Gastrointestinal Medical Oncology, Harbin Medical University Cancer Hospital, Harbin, Heilongjiang 150081, P.R. China; 2 Department of Medical oncology, Xianyang Central Hospital, Xianyang, Shanxi 712000, P.R. China; 3 Department of acupuncture, 1st Affiliated Hospital of Harbin Medical University, Harbin, Heilongjiang 150000, P.R. China

**Keywords:** Naringenin, colorectal cancer, high-fat diet, gut microbiota, IL-6/STAT3 pathway

## Abstract

Colorectal cancer (CRC) is a worldwide health issue. It causes illness and death in millions of people each year. A positive correlation has been observed between the intake of dietary fat and the development of CRC. The composition of gut microbiota exhibits a significant correlation with pathophysiologic processes in intestine. Clinical treatment remains inadequate due to the complex pathogenic mechanisms of CRC triggered by a high-fat diet (HFD). Naringenin, a flavonoid from grapefruit, has anti-cancer activity. Our findings suggest that naringenin enhances gut microbiota diversity by increasing the abundance of beneficial bacterial species while reducing opportunistic pathogenic bacteria. The fecal microbiota transplantation assay (FMT) demonstrated that the anti-HFD-CRC activity of naringenin depended on the gut microbiota. Furthermore, naringenin antagonized the IL-6/STAT3 pathway. These results suggest that naringenin may be a potential treatment for HFD-CRC.

## Introduction

Colorectal cancer (CRC) is a malignant tumor that occurs within various countries and is the leading cause of cancer deaths [1]. In addition to an aging population and insufficient levels of physical activity, obesity and the dietary habits of developed countries increase the risk of CRC. Therefore, maintaining regular exercise, balanced nutrition and a healthy lifestyle is essential to reduce the risk of CRC [2]. High fat, red meat, and processed meat intake are significant causative factors. In contrast, intake of dietary fiber may partially offset the adverse effects of meat, such as shortening intestinal transit time and diluting carcinogenic compounds [3]. Research has indicated that a high-fat diet (HFD) increases colorectal tumor incidence by inducing gut microbiota dysbiosis and intestinal barrier dysfunction in mice [4]. As the economy grows, the fat in the diet increases. Therefore, it is imperative to conduct comprehensive research on the prevention of high-fat diet-induced colorectal cancer (HFD-CRC). The HFD-associated gut microbiota enhances colon cell proliferation and promotes tumorigenesis. Depletion of the microbiota leads to a heightened occurrence and frequency of colonic tumors in certain mice models of CRC [5].

In recent years, Chinese medicine and the chemical composition of agricultural products have attracted much attention for its exploration and research on tumor prevention and treatment [6]. Flavonoids are naturally occurring compounds found widely in plants and various leaves, fruits, and dry fruit components [7]. They have important biological properties including sterilization, anti-infective effects, anti-allergic activity, antioxidant capabilities against free radicals, and anti-tumor effects. The anticancer effects of flavonoids on CRC cells are believed to stem from their capacity to influence critical signaling pathways involved in cell growth and survival. For example, flavonoids can inhibit the activity of enzymes that promote cell proliferation, and they can also induce apoptosis, which is the programmed death of cancer cells [8]. The relationship between inflammation and cancer development has been confirmed by extensive data [9]. Flavonoids have anti-inflammatory properties [10]. Therefore, they are of interest in nutrition and pharmacology [11]. In human breast cancer cells, flavonoids have been shown to inhibit cell proliferation and promote apoptosis and autophagy [12].

Naringenin is a flavonoid polyphenol found mainly in citrus fruits such as grapefruit, as well as medicinal plant-derived foods. Naringenin attenuates the expression of pro-inflammatory factors in oxidized low-density lipoprotein-treated human umbilical vein endothelial cells and restores endothelial barrier integrity, suggesting that naringenin may have a therapeutic role in endothelial injury-related diseases [13]. Naringenin has been widely used to prevent cardiovascular diseases such as atherosclerosis, hypertension, and arrhythmia [14-16]. Naringenin exerts anticancer effects by inducing apoptosis, promoting cell cycle arrest, and regulating multiple signaling pathways, including Wnt/β-catenin, PI3K/Akt, NF-κB pathways [14]. Naringenin inhibits various cytokines and reactive oxygen species (ROS) generators by modulating nuclear factor κB (NF-κB) activity [17]. Naringenin treatment not only leads to a dose-dependent reduction in tumor incidence and tumor load but also alters inflammation-related biochemical indices and antioxidant parameters, which are necessary for anticancer activity. qRT-PCR confirmed the action of naringenin mediated through the mitochondrial pathway [18]. Studies have shown that naringenin disrupts the cell cycle, increases the expression of pro-apoptotic genes, down-regulates the expression of anti-apoptotic genes, and decreases the expression of pro-survival signaling pathways, resulting in apoptosis and chemo sensitization of colorectal cancer cells [19, 20]. However, the role of naringenin in HFD-CRC and the gut microbiota remains ambiguous.

We hypothesized that naringenin may inhibit HFD-CRC by improving the gut microbiota and gut barrier integrity. We treated C57BL/6 mice with AOM/DSS and HFD to validate the inhibitory impact of naringenin on HFD-CRC tumorigenesis. 16S rRNA sequencing was conducted to assess the regulatory impacts of NAR on the gut microbiota. In addition, this study validated the function of the gut microbiome in tumorigenesis by C57BL/6 gut microbiome depletion model mice and FMT. The results suggest that NAR has potential in the treatment of HFD-CRC.

## Materials and Methods

### Animal Experiments

C57BL/6 male mice were obtained from the Animal Center of Harbin Medical University. Mice were placed in a standardized animal husbandry laboratory with access to adequate food and water. The Ethics Committee approved all animal studies for Animal Experiments at Harbin Medical University (Approval No. KY2023-58).

After 2 weeks of adaptation, the mice were assigned to three experimental groups (*n* = 8 per group): the normal diet (ND) group, the High-fat diet (HFD) group, the HFD combined with NAR (HFD+NAR) group. Each mouse was injected intraperitoneally with azoxymethane (AOM) at a dose of 10 mg/kg once (Aladdin, China), after which they underwent three cycles of consuming 2% dextran sulfate sodium (DSS) for a duration of 7 days, interspersed with 14 days of water consumption. Normal feed was given to ND group and HFD was given to the remaining two groups. HFD+NAR group was treated with NAR (100 mg/kg, Aladdin). NAR was administered via gavage in a solution of 0.5% sodium carboxymethyl cellulose, (CMC-Na) once daily for 10 weeks at the start of modeling. As a control, both the ND and HFD groups received the same dose of CMC-Na orally once daily for 10 weeks. At the conclusion of the experiment, all mice were subjected to anesthesia via subcutaneous administration of 1% sodium pentobarbital to obtain serum and killed to obtain colon tissue. Collection of colon tissue for histopathological, immunohistochemical studies and Western Blot. Cardiac blood (2~3 ml) was taken after anesthesia for biochemical measurements. Feces were collected and frozen for gut microbiota analysis.

### Fecal Microbiota Transplantation (FMT) Experiment

C57BL/6 male mice, aged 6 weeks, were administered a cocktail of antibiotics consisting of ampicillin (0.5 g/l), metronidazole (1 g/l), vancomycin (0.5 g/l), and neomycin (0.5 g/l) (Sigma-Aldrich, USA) for a duration of two weeks in order to create a pseudo-bacteria-free mouse model prior to the implementation of fecal microbiota transplantation (FMT). Two of these groups received gavage treatment with fecal samples obtained from HFD group or HFD+NAR group respectively. Another group was administered phosphate-buffered saline (PBS). 100 mg of feces was homogenized in 1 ml of PBS. Subsequently, mice were garaged with 0.2 ml of this mixture 3 times per week. After taking serum and feces, mice were executed to obtain colon tissue.

### Cell Culture and Treatment

HCT 116 and SW 480 cell lines were cultured in DMEM/F-12 medium enriched with 10% fetal bovine serum (FBS), 100 U/ml penicillin, and 100 mg/ml streptomycin. The cells were cultured under controlled conditions of temperature and humidity. Naringenin was solubilized in dimethyl sulfoxide (DMSO) prior to the administration of treatment to the cells. HCT 116 and SW 480 cells were inoculated in 96-well plates and incubated overnight. Cells were treated with different concentrations of naringenin for 24 h. The cells were incubated with MTT solution for 2 h. Absorbance was read at 570 nm to determine the formation of post larvae.

### Hematoxylin and Eosin (H&E) Staining

Fresh colon tissue was fixed and embedded, and 5-μm sections were prepared. Then dewaxing and rehydration were performed. Perform hematoxylin and eosin staining for 3-5 min. Images were obtained utilizing an Olympus BX 51 microscope, and cross-sectional areas were quantified using IMT iSolution DT 9.2 software. A microscopic examination of the sections was performed by a pathologist unaware of this experiment.

### Immunohistochemical Analysis

Colon tissues embedded in paraffin and mounted on glass slides underwent a series of preparatory steps, including deparaffinization, antigen-retrieved, blocked, and incubated with an Abs against Ki67 (Proteintech Group, China), Zo-1 (Affinity Biosciences, China), and Occludin (Proteintech) overnight at 4°C. The sections were subjected to incubation with an anti-rabbit secondary antibody for 1 h at room temperature and incubated with avidin-biotin complexes. Staining was performed with 3,3-diaminobenzidine according to the manufacturer’s protocol. Restraining was performed using Mayer hematoxylin (Affinity). Stained slides were examined utilizing an Olympus BX 51 microscope.

### Enzyme-Linked Immunosorbent Assay (ELISA) Analysis

ALT, AST, lipopolysaccharide (LPS) levels and CK-MB were measured using kits from Jiangsu Jingmei (China). Serum levels of inflammation were evaluated utilizing the appropriate assay kits, which included IL-1β, IL-6, and TNF-α (Jianglaibio, China).

### Oxidative Stress Marker Assay

Oxidative stress markers for each group of colon tissues were assayed according to the kit requirements. The levels of superoxide dismutase (SOD, Beyotime Biotechnology, China), catalase (CAT, Beyotime), malondialdehyde (MDA, Beyotime), reduced glutathione (GSH, Beyotime), and oxidized glutathione (GSSG, Beyotime). The expression of glutathione peroxidase (GSH-Px, Beyotime) was evaluated utilizing the NADPH-dependent total glutathione peroxidase assay kit (Beyotime).

### Western Blot Analysis

Western blotting analysis was performed in accordance with established standard operating procedures. Total proteins were extracted from tissues using radioimmunoprecipitation (RIPA, Seven, China). Protein concentration measurements using BCA kits (Beyotime). Total protein was isolated using sodium dodecyl sulfate–polyacrylamide gel electrophoresis (SDS-PAGE) (Beyotime) and subsequently transferred to polyvinylidene difluoride (PVDF) membranes (Beyotime). Incubate the membrane with milk for 2 h. They were used for simultaneous detection with different primary antibodies:β-actin (Proteintech), GAPDH(Aabcam, UK), CLAUDIN-3(Cell Signaling Technology, USA),occluding (CST), ZO-1(CST), IL-1β (Affinity), IL-6(Affinity), stat-3(Proteintech), p-stat-3(Affinity), β-catenin (Proteintech), E-cadherin(CST) and TNF-α(Affinity). After overnight at 4°C, incubation with secondary antibody was performed for 2 h. The membrane was washed and incubated for protein band intensity and quantified separately in ImageJ software.

### Gut Microbiota Analysis

Genomic DNA from bacteria was isolated from fecal specimens. Subsequently, the V3-V4 regions of the 16S rRNA were amplified through polymerase chain reaction (PCR) utilizing the forward primer and the reverse primer. The products of the PCR amplification were subsequently purified via electrophoresis on a 2% agarose gel, ensuring that the electrophoresis tank was maintained in a clean condition and that the electrophoresis buffer was replaced as necessary.

### Statistical Analysis

Statistical analyses were conducted utilizing GraphPad Prism software, version 10.1.2, developed by GraphPad Software, USA. A one-way ANOVA was utilized for the comparison of more than two groups. Unpaired *t*-tests with two-tailed distributions were used when comparing two sets of data. Detailed information on all experimental replications is provided in the respective figure legends. Statistical significance was determined at *p* < 0.05 (*) and *p* < 0.01 (**), following multiple hypothesis correction. In the analysis of diversity, the Kruskal-Wallis test function within the R programming language was employed to compare samples across multiple groups. Additionally, beta diversity was assessed using QIIME software.

## Results

### Naringenin Inhibits HFD-Associated Colorectal Tumorigenesis and Progression in Mice

**Fig. 1:**
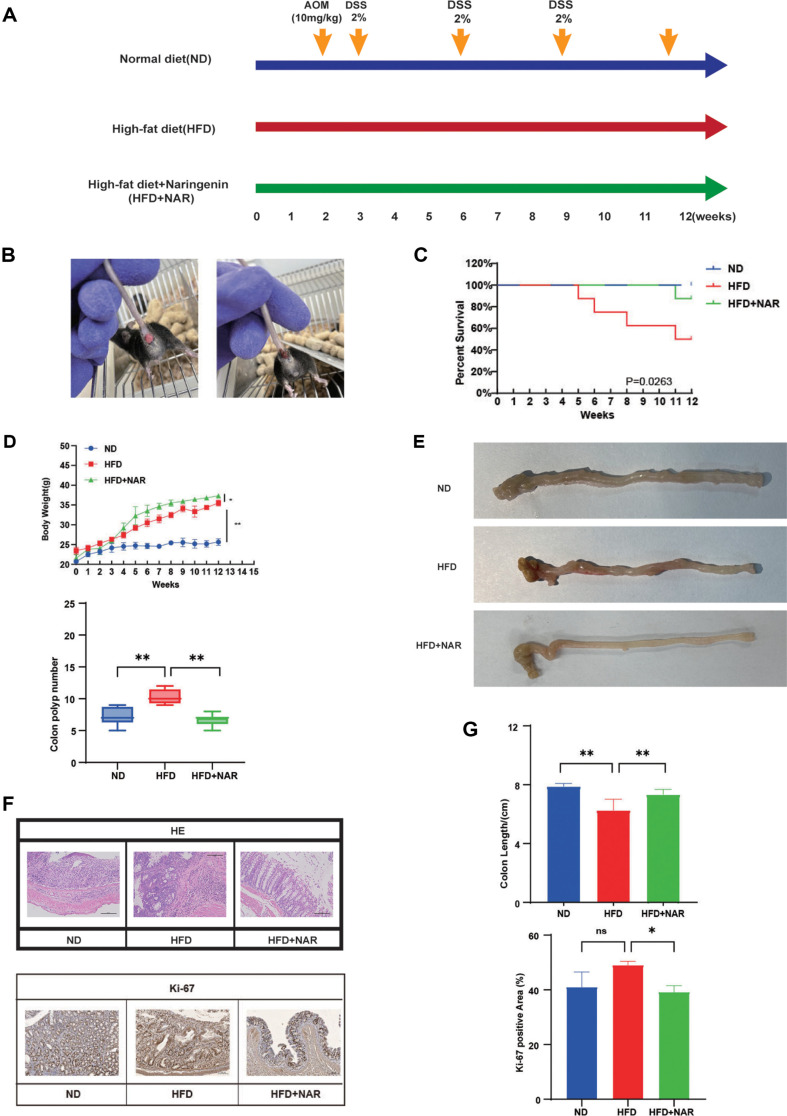
NAR inhibits HFD-associated colorectal tumourigenesis in mice. (**A**) NAR protocol in AOM/DSS mice fed HFD; (**B**) Anal swelling, bleeding, and anorectal prolapse in AOM/DSS model mice; (**C**) The survival rate of mice in three groups; (**D**) Curves of weight change in the three groups and the number of colon polyps in the three groups; (**E**) Image of the colon at the time of execution; (**F**) H&E staining for colon (scale bar: 100 μm) and Colorectal section immunohistochemistry (IHC) staining showing Ki-67 expression: bar = 20 μm; (**G**) Colon length and Ki-67 positive area (*p < 0.05, **p < 0.01).

To explore how naringenin affects a HFD-associated colorectal tumors, we followed the experimental procedure described in Methods and Materials to assess the potential effects (Fig. 1A). At the conclusion of the experiment, characteristic anorectal features in the mice were documented, including swelling, bleeding, and anorectal prolapse, which are indicators of success in the AOM/DSS-induced CRC model (Fig. 1B). The survival rate of mice treated with naringenin was much higher than that of the control group mice receiving only HFD (Fig. 1C). Meanwhile, the mice's body weights were recorded weekly throughout the experiment. At week 12, the body weights of the mice subjected to a HFD exhibited a significant increase in comparison to the group that was fed a normal diet (ND) (Fig. 1D). Compared with HFD-fed mice, there are fewer polyps and longer colon length in NAR-fed mice (Fig. 1E-1G). Histological examination with H&E staining showed that HFD caused a marked increase in tumor cells in intestinal tissues and a notable infiltration of inflammatory cells in the submucosal layer. In contrast, naringenin treatment significantly attenuated these changes (Fig. 1H). We detected the concentrations of ALT and AST in mice serum, and in this experiment, we did not find any significant damage to the liver function of mice caused by naringenin (Fig. S1). On the contrary, naringenin reduced the degree of damage to the liver and heart. Immunohistochemistry results showed more Ki-67 positive cells in colon sections of mice fed HFD compared to ND. The findings indicate that the proliferation of colonic epithelial cells was markedly elevated in mice subjected to a HFD. Nevertheless, the administration of naringenin resulted in a substantial decrease in the quantity of Ki-67 positive cells (Fig. 1I).

### Naringenin Alleviates HFD-Induced Impairment of Intestinal Barrier Function

**Fig. 2:**
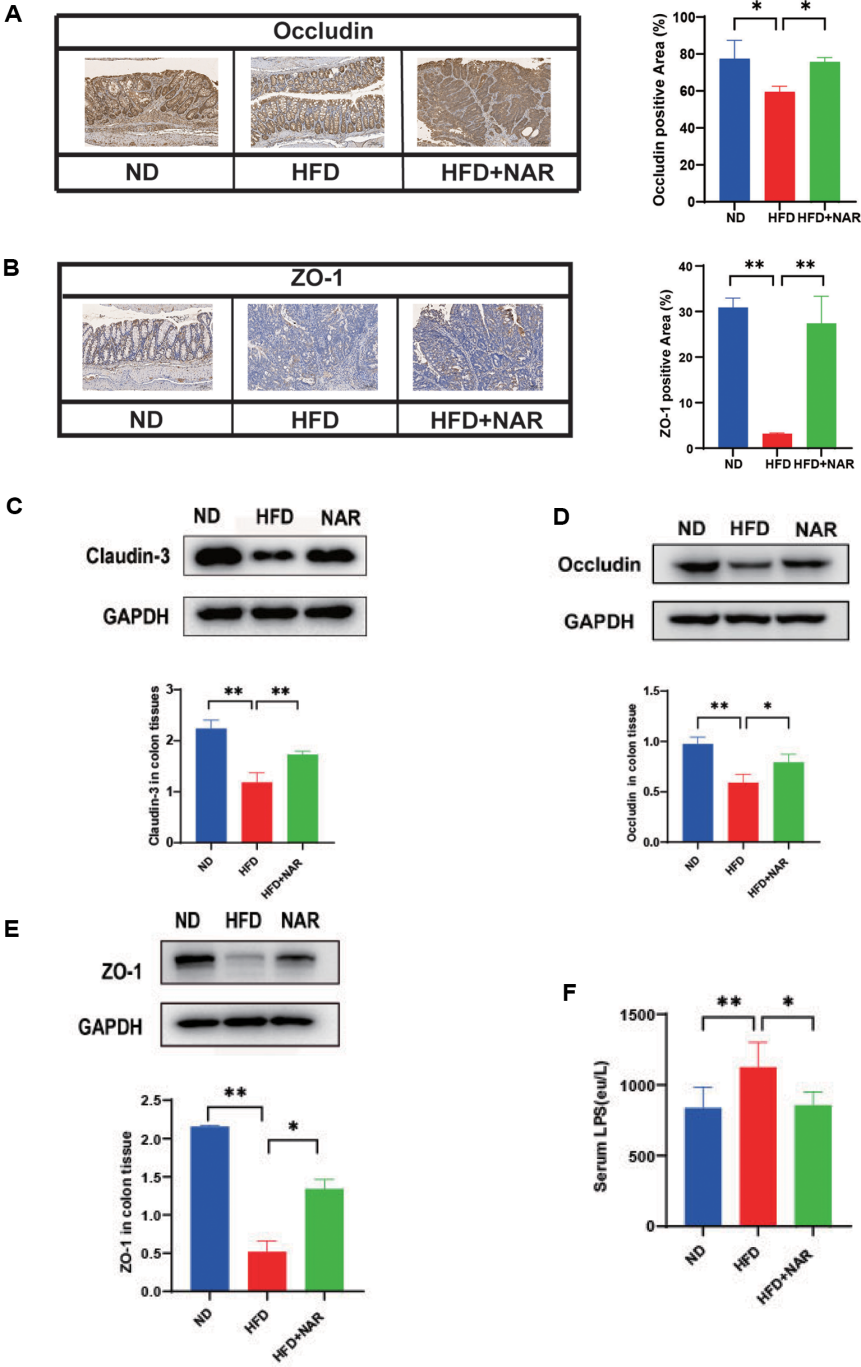
Nar alleviates HFD-induced impairment of intestinal barrier function. (**A**) IHC staining of colorectal sections, showing occludin expression: bar = 100 μm; (**B**) IHC staining of colorectal sections, showing ZO-1 expression: bar = 100 μm; (**C**) Expression of claudin-3 in colon tissue; (**D**) Expression of occludin in colon tissue; (**E**) Expression of ZO-1 in colon tissue; (**F**) Serum LPC concentrations in the three groups of mice. (*p < 0.05, **p < 0.01).

Preservation of the intestinal barrier's integrity is essential for the maintenance of homeostasis. The secretion of inflammatory cytokines has the potential to compromise the structural integrity of the intestinal barrier, resulting in heightened intestinal permeability [21]. Lipopolysaccharide (LPS) and other water-soluble molecules can cross the intestinal mucosa via the paracellular pathway [22]. Studies have shown that a high-fat diet can compromise the integrity and function of the intestinal barrier [23]. We conducted an analysis of the levels of colonic tight junction proteins, including occludin, claudin-3, and zona occludens-1 (ZO-1), as well as serum LPS levels. Immunohistochemical analysis showed that naringenin treatment increased the expression of ZO-1 and occludin (Fig. 2A and 2B). Similarly, in colonic tissue, mice fed hfd had significantly lower levels of tight junction proteins compared to mice fed ND. Expression levels of claudin-3, occludin, and ZO-1 were significantly increased after naringenin treatment, as shown in Western blotting (Fig. 2C-2E). Meanwhile, naringenin reduces serum LPS elevation induced by high-fat feeds (Fig. 2F). These results suggest that naringenin mitigates the impaired intestinal barrier function of HFD feeding.

### Naringenin Inhibits Inflammatory Cytokines and Oxidative Stress in HFD-Fed Mice

**Fig. 3:**
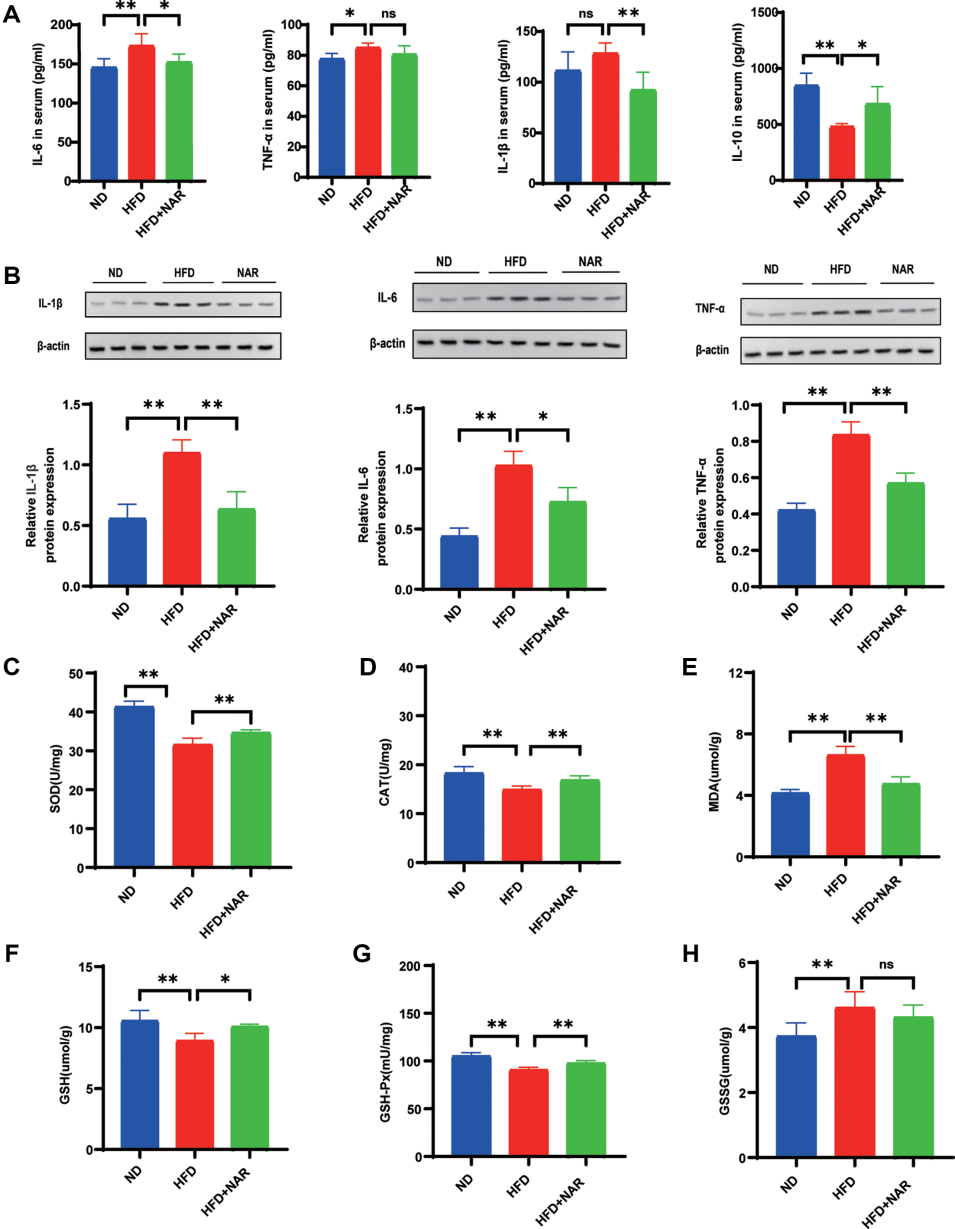
NAR inhibits inflammatory cytokines and oxidative stress in AOM/DSS-treated HFD-fed mice. (**A**) Levels of inflammatory cytokines (IL-6, TNF-α, IL-1β, IL-10) in serum; (**B**) Levels of inflammatory cytokines (IL-1β, IL-6, TNF-α) in colon tissue; (**C-H**) Colonic levels of SOD (**C**), MDA (**D**), CAT (**E**), GSH (**F**), GSH-Px (**G**) and GSSG (**H**).

Dysbiosis of the intestinal microbiota is significantly associated with inflammation, which serves as a connection between oncogenic factors and the process of tumorigenesis. To test the effect of naringenin on inflammation, we examined inflammatory cytokines in serum and colon tissue. Naringenin attenuated the elevation of pro-inflammatory factors in the serum of the HFD group. In contrast, the concentration of the anti-inflammatory factor IL-10 was significantly lower in the HFD group than in the ND group (ND). However, the IL-1β, IL-6, and TNF-α concentrations in serum were reduced after naringenin treatment in HFD-fed mice. Similarly, serum IL-10 levels increased significantly after naringenin treatment (Fig. 3A). In addition, western blot analysis showed elevated levels of inflammatory factors in the colonic tissues of mice on HFD compared to mice on ND. Notably, naringenin significantly reduced the expression of proinflammatory factors in the colonic tissues of hfd-fed mice (Fig. 3B).

Mice in the HFD-fed group had lower levels of SOD, GSH, GSH-Px, and CAT. And they had higher levels of MDA and GSSG. In addition, the colonic tissues of the NAR-administered group showed substantially higher SOD, CAT, GSH, and GSH-Px expression than that of the HFD-fed group. The levels of malondialdehyde (MDA) were found to be elevated in the NAR-fed compared to the HFD group (Fig. 3C-3H). These results suggest that naringenin can inhibit HFD-associated colorectal tumorigenesis by suppressing inflammatory and oxidative stress.

### NAR Inhibits the IL-6/STAT 3 Signaling Pathway

**Fig. 4:**
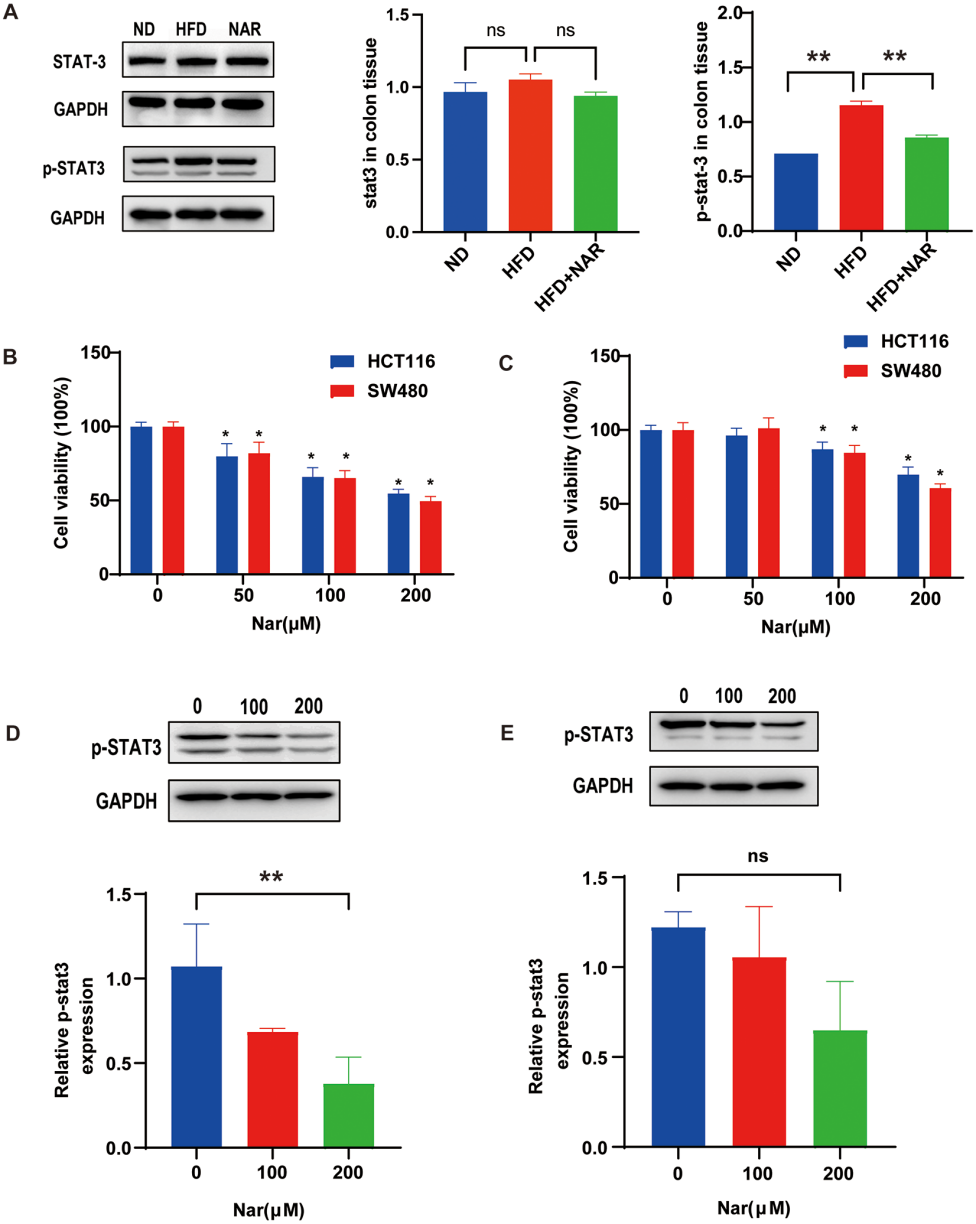
NAR inhibits the IL-6/STAT 3 signaling pathway. (**A**) Expression of STAT3 and p-STAT3 in the colon tissues; (**B**) HCT 116 and SW 480 cells are treated with the indicated concentrations of NAR for 24 h. Cell viability is measured using the MTT assay system and expressed as % cell growth compared to cells without NAR; (**C**) HCT 116 and SW 480 cells are treated with indicated concentrations of NAR and exogenous IL6 for 24 h. Cell viability is measured using the MTT assay system; (**D**) HCT 116 cells are seeded overnight and then treated with the indicated concentrations of NAR for 24 h. Expression of p-STAT3 in cells. (**E**) HCT 116 cells are seeded overnight and then treated with the indicated concentrations of NAR and IL-6 for 24 h. Expression of p-STAT3 in cells.

The IL-6/STAT3 signaling pathway is known to be involved in the pathogenesis of colitis and related cancers [24, 25]. To further explore the mechanism of HFD-CRC inhibition by naringenin, we analyzed the levels of colonic STAT3 and phosphorylated STAT3 (p-STAT3). Naringenin effectively inhibited the expression of P-STAT3 that was elevated in the HFD group (Fig. 4A). We verified *in vitro* whether exogenous IL-6 or IL-1β rescued the antitumor effects of NAR. HCT 116 and SW 480 cell lines were treated with 0, 50, 100 and 200 μM naringenin for 24 h. Subsequent analyses were conducted using MTT assays and Western blotting techniques. After 24 h of treatment with naringenin, the viability of HCT 116 and SW 480 cells was significantly reduced dose-dependent, as determined by the MTT assay. In HCT116 and SW480 cells, naringenin inhibited cell viability by 7% and 9% at 50 μM, 24% and 28% at 100 μM, and 45% and 50% at 200 μM, respectively (Fig. 4B). The addition of 50 ng/ml exogenous IL-6 significantly rescued the reduced cell viability after treatment with naringenin (7% and 9% at 50 μ M, 14% and 16% at 100 μM, and 31% and 40% at 200 μM respectively) (Fig. 4C). In contrast, adding 50 ng/ml of IL-1β failed to rescue cell viability significantly (Fig. S2). This suggests that IL-6 is essential in naringenin inhibition of colorectal cancer. To verify whether naringenin downregulates the level of p-STAT3, HCT 116 cells were treated with 0, 100, and 200 μM NAR for 24 h, followed by Western blotting. The results showed that NAR dose dependently reduced the level of p-STAT3 protein in HCT 116 cells (Fig. 4D). Exogenous IL-6 rescued the reduced level of p-STAT3 protein expression (Fig. 4E).

### Naringenin Modulates the Gut Microbiota in HFD-Fed Mice

**Fig. 5:**
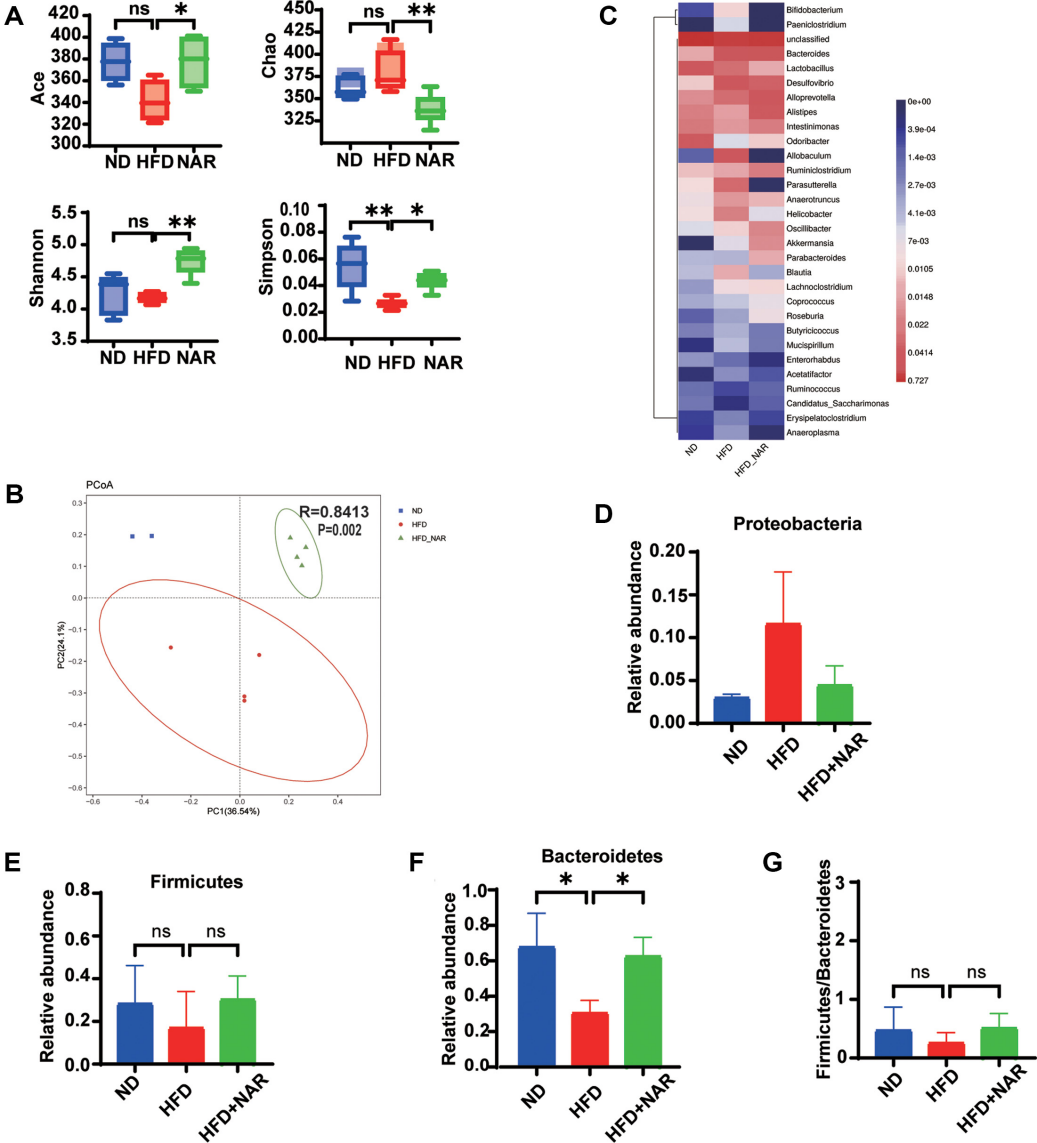
NAR modulates the gut microbiota in AOM/DSS-treated mice. (**A**) Richness indices (Chao1 and Ace) and diversity indices (Shannon and Simpson) of gut microbiota among three groups: (**B**) Principal component ordination analysis of the gut microbiota among the three groups. (**C**) Heat map of the relative abundance of the first 30 enriched bacterial OTUs between the three groups; (**D-G**) Relative abundance of Proteobacteria (**D**), Firmicutes (**E**), Bacteroidetes (**F**), and Firmicutes / Bacteroidetes (**G**) in fecal samples. (*p < 0.05, **p < 0.01)

The alteration of gut microbiota composition presents a promising therapeutic strategy. Therefore, we analyzed fecal samples for 16S rRNA sequencing. For α-diversity, Naringenin significantly counteracted the HFD-induced reduction in bacterial abundance, as shown by the change in the AC, Shannon, Simpson, and Chao indices (Fig. 5A). For β-diversity, principal coordinate analysis (PcoA) revealed different patterns of gut microbiota in the three groups of mice, suggesting that naringenin treatment can alter the composition of the gut bacterial community in mice (Fig. 5B). Bioinformatics analysis showed that at the phylum level, the top three were Bacteroidetes, Firmicutes, and Proteobacteria (Fig. 5C). The abundance of the potentially pathogenic bacteria Proteobacteria and Firmicutes was higher in the HFD group than in the ND group (Fig. 5D-5E), the beneficial bacteria Bacteroidetes was lower than in the normal diet (ND) group (Fig. 5F) and naringenin reduced the ratio of Firmicutes / Bacteroidetes (F/B) in mice (Fig. 5G). Although not statistically significant possibly due to insufficient sample size. Our subsequent research will expand the sample size to further verify this result.

**Fig. 6:**
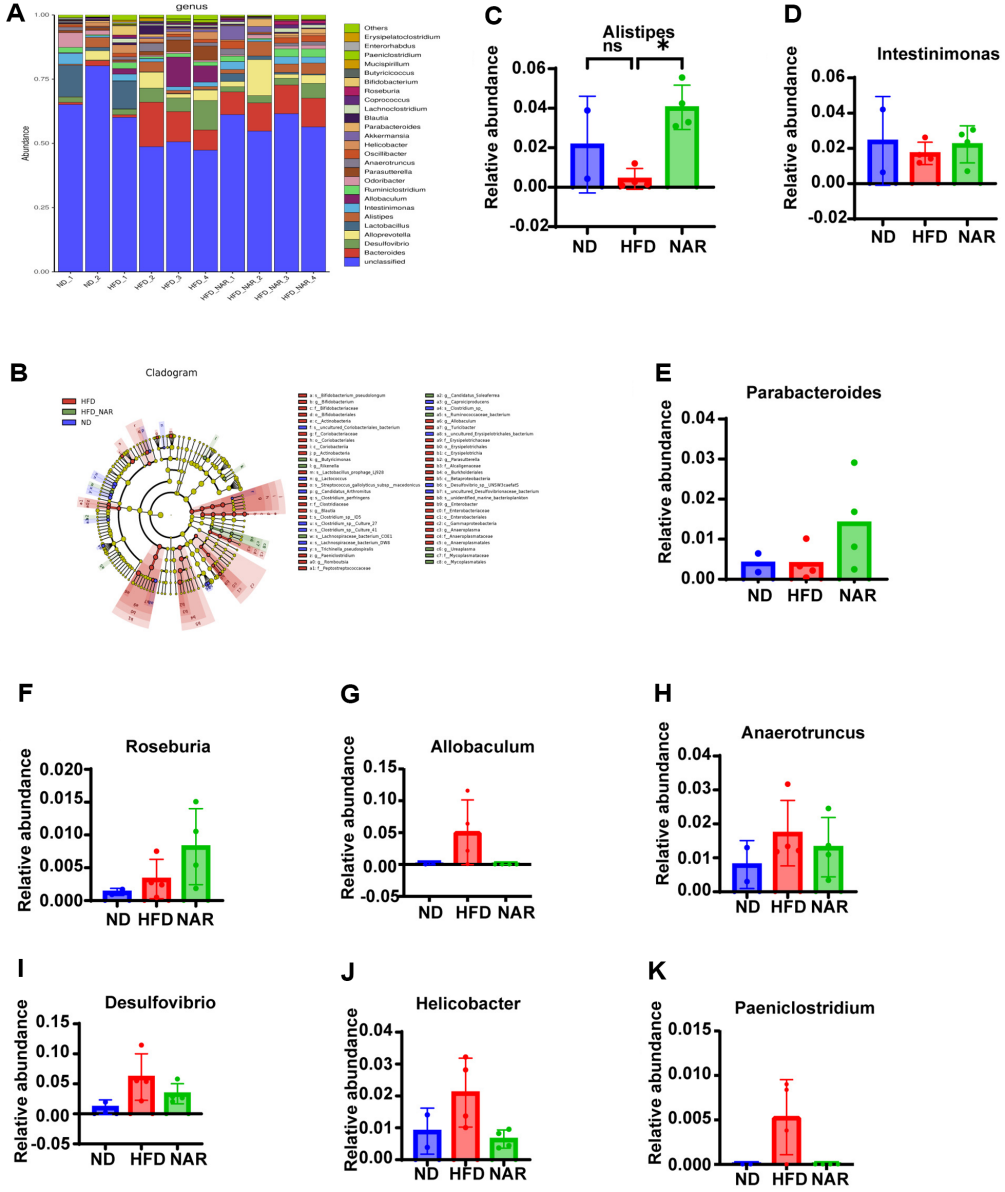
NAR modulates the gut microbiota in AOM/DSS-treated mice. (**A**) Bacterial taxonomic profiling at the genus level among samples; (**B**) LEFse analysis of three groups. (**C-K**) Relative abundance of gut microbiota at the genus level in all groups; relative abundance of (**C**) Alistipes, (**D**) Intestinimonas, (**E**) Parabacteroides, (**F**) Roseburia, (**G**) Alloprevotella, (**H**) Anaerotruncus, (**I**) Desulfovibrio, (**J**) Helicobacter, and (**K**) Paeniclostridium. in three groups. (*p < 0.05, **p < 0.01)

The intestinal flora among the groups was highest in the genere *Bacteroides*, *Desulfovibrio*, *Alloprevotella*, *Lactobacillus*, *Alistipes* and *Inteinimonas* (Fig. 6A). The administration of naringenin effectively reversed the alterations in the relative abundance of *Alistipes*, bringing it back to the levels observed in the normal diet group (Fig. 6C). Naringenin administration increased levels of several low-beneficial genera in HFD, including *Intestinimonas*, *Parabacteroides*, and *Roseburia* (Fig. D-F). In addition, NAR-fed increased the levels of several harmful genera that were induced to be elevated by HFD, including *Allobaculum*, *Anaerotruncus*, *Desulfovibrio*, *Helicobacter*, and *Paeniclostridium*. However, some did not reach statistical significance (Fig. 6G-6K). Next, the Cladogram revealed the enrichment of specific taxa in the ND group, such as *Coriobacteriales*, *Lactococcus*, and *Candidatus*_*Soleaferrea*. In contrast, *Bifidobacteriales* and *Coriobacteriia* were enriched in the HFD group. The naringenin treatment induced a trend towards increased *Rikenellaceae*, *Lachnospiraceae*_bacterium_COE1, and *Ruminococcaceae*, which are known for their beneficial roles in mitigating inflammation, obesity, intestinal disorders, and immune disorders (Fig. 6B).

### FMT Recapitulates Naringenin’s Inhibitory Effect on Colorectal Tumorigenesis in Mice

**Fig. 7:**
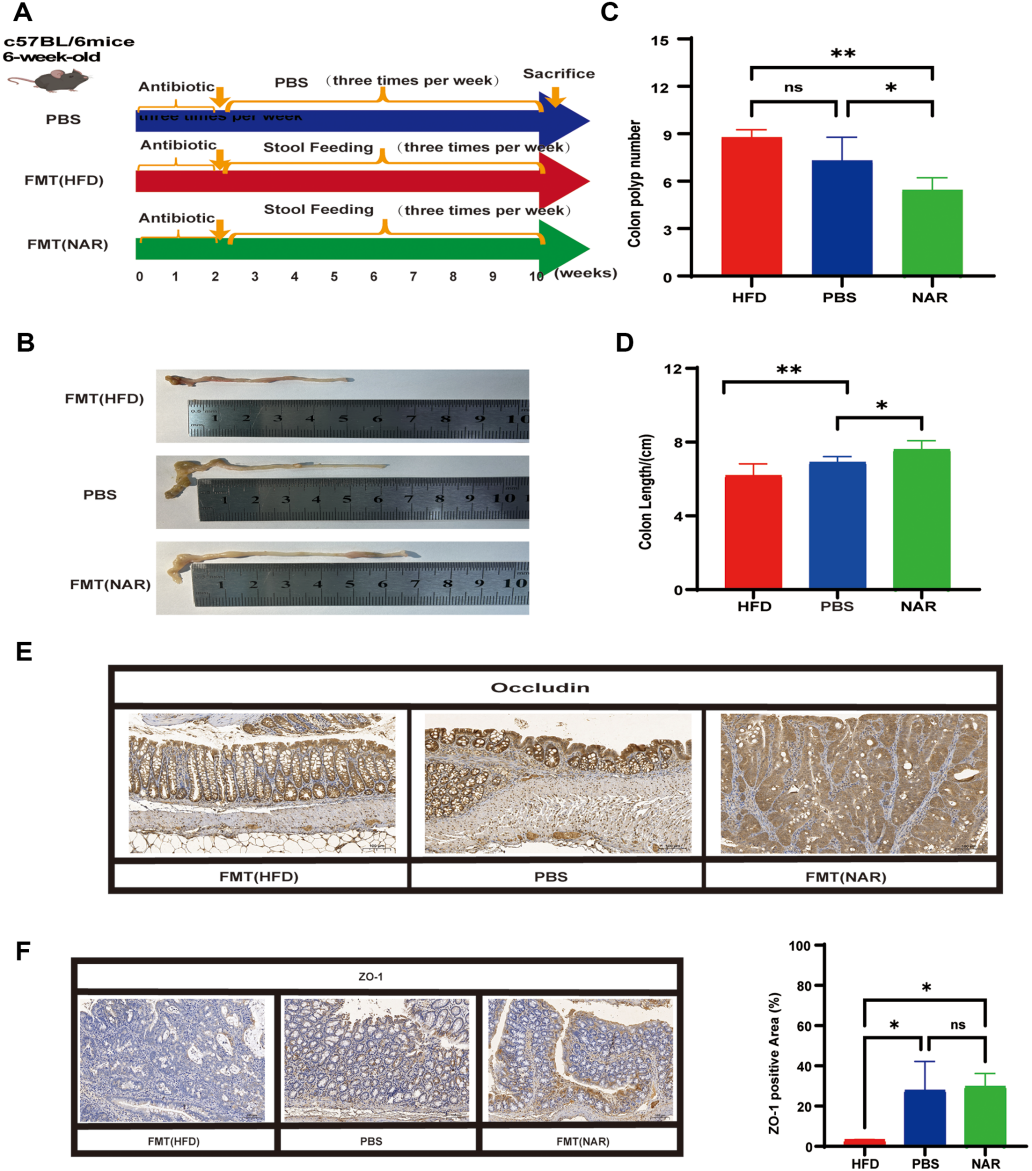
FMT recapitulates NAR's inhibitory effect on colorectal tumorigenesis in C57BL/6 mice. (**A**) Experimental design of antibiotics in fecal-fed C57BL/6 mice (**B**) Image of the colon at the time of execution (**C**) The number of colon polyps (**D**) Colon length (**E**) IHC staining of colorectal sections, showing occludin expression: bar = 100 μm (**F**) IHC staining of colorectal sections, showing ZO-1 expression: bar = 100 μm. (*p < 0.05, **p < 0.01).

To further validate the role of naringenin regulated gut microbiota in the development of HFD-CRC, we tested its effect in sterile C57BL/6 model mice. We treated colorectal cancer mice with an antibiotic mixture for 2 weeks to reduce their endogenous flora and establish a pseudo sterile mouse model. Then, fecal samples from naringenin fed (FMT-NAR) mice were orally administered three times a week for 10 weeks, while fecal samples from HFD fed (FMT-HFD) mice were used as the control group and PBS was used as the blank control (Fig. 7A). Our findings suggest that naringenin-treated fecal samples showed a reduction in the number of polyps and attenuated the reduction in colon length (Fig. 7B and 7C). We also examined the expression of Ki-67, Occludin and ZO-1 in colon tissues by immunohistochemistry. In colonic tissues, Occludin was elevated in FMT-NAR mice and decreased in FMT-HFD mice. ZO-1 was elevated in FMT-NAR mice compared to the FMT-HFD group. Although ZO-1 levels were not significantly different in FMT-NAR mice compared to PBS mice, ZO-1 levels in fecal samples from HFD mice were reduced compared to the PBS group (Fig. 7D-7E). Interestingly, there was no difference in Ki-67 levels (Fig. S3).

**Fig. 8:**
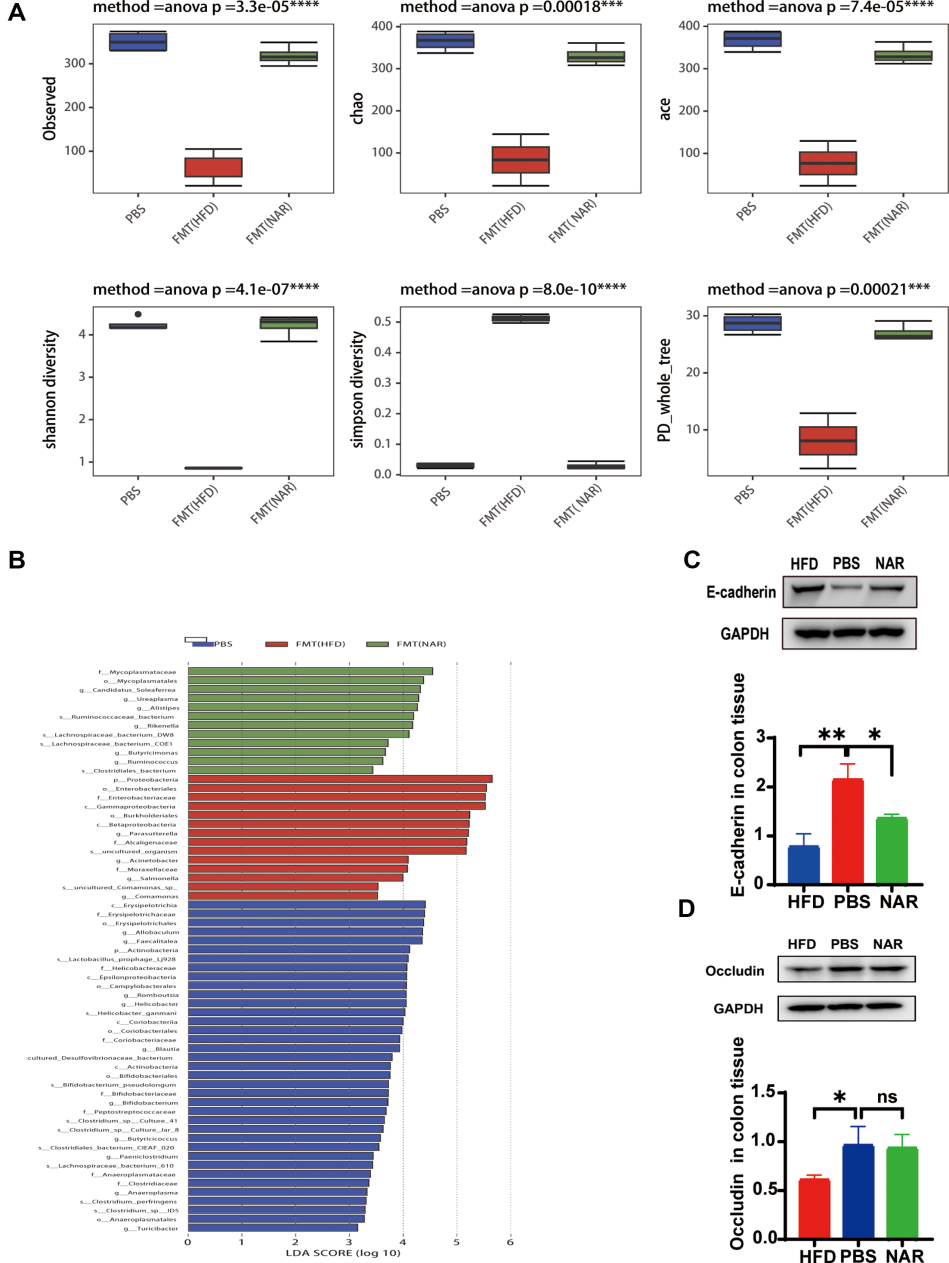
FMT recapitulates NAR's inhibitory effect on colorectal tumorigenesis in C57BL/6 mice. (**A**) **α** diversity of the microflora (**B**) LEFse analysis of three groups (**C**) Expression of E-cadherin in colon tissue (**D**) Expression of occludin in colon tissue. (*p < 0.05, **p < 0.01)

We employed 16S rRNA sequencing to analyze the structure of the gut microbiota following FMT treatment, as well as to determine whether the gut microbiota was successfully colonized after FMT. Bacterial abundance and diversity were observed to be lower in the FMT-HFD than in the FMT-NAR as well as the PBS groups (Fig. 8A). Next, LEFSe analysis revealed the enrichment of specific taxa in the FMT-HFD group, such as p__Proteobacteria, Enterobacteriales, Gammaproteobacteria, and Burkholderiales. In contrast, the FMT-NAR induced an increase in the number of *Clostridiales*_*bacterium*, Lachnospiraceae_bacterium_DW8, _Ruminococcaceae_bacterium known for their beneficial role in reducing inflammation and obesity (Fig. 8B). These results indicate that NAR exerts inhibitory effects on HFD related colorectal tumors through the gut microbiota. Meanwhile, in colonic tissues, E-cadherin and occludin protein expression were significantly higher in NAR-FMT mice and PBS mice compared to mice in the HFD-FMT group (Fig. 8C and 8D). At the phylum level, the FMT-HFD group had the highest levels of proteobacteria flora, while the FMT-NAR group had significantly lower levels. Significant differences were also found at the genus level. The flower diagram showed that the intestinal microbiota species of the FMT-NAR group were much higher than those of the FMT-HFD group (Fig. S4).

## Discussion

Epidemiological studies have shown that HFD typically leads to obesity, elevating the likelihood of colorectal cancer (CRC) by 30 to 70% [26]. A high-fat diet (HFD) exacerbates intestinal inflammation and compromises the integrity of the intestinal barrier through the modulation of gut microbiota and associated metabolites, thereby facilitating the development of colorectal tumors [27]. The gut microbiota contributes beneficially to both the diagnosis and treatment of CRC. Bacteria can diagnose marker diseases or act as modulators of chemotherapy and immunotherapy. In addition, metabolites of the gut microbiota play a role in interactions with CRC cells [28]. Colitis is a risk factor for colorectal cancer. The azomethane dextran sulfate sodium (AOM/DSS) model is a commonly used experimental model for colorectal cancer [29]. The model is highly reproducible, affordable and practical [30]. Studies in this model have emphasized the important role of colitis in colorectal cancer progression, revealing some of the mechanisms of colon carcinogenesis associated with intestinal inflammation, such as inflammatory factors [5, 10, 31]. Consequently, there is a pressing necessity to establish effective and safe therapeutic approaches for HFD-CRC.

Our study showed that naringenin altered the structure of gut microbes in the HFD group of mice, reducing the levels of harmful bacteria by changing the number of OTUs, alpha diversity and species variety. Naringenin has been shown to inhibit inflammatory and oncogenic pathways within the intestine, while also markedly reducing epithelial barrier damage in a mouse model of CRC induced by a high-fat diet. The transplantation of fecal matter from mice fed a NAR diet into microbiota-depleted models of CRC suppressed the disease. To our knowledge, this is the first study to propose that naringenin inhibits CRC by regulating gut microbiota. Findings from the NHANES cohort suggest that higher flavonoid intake slows systemic biological aging, cardiovascular and hepatic biological aging [32]. A study followed up 1,779 participants for a median of 11.8 years. In terms of overall function, verbal and visual memory, a significant trend of slower cognitive decline was observed among participants with higher intakes of flavanols. Higher flavanol intake was beneficial for language learning [33]. Therefore, we speculate that the long - term use of flavonoids still has considerable therapeutic effects on diseases and causes no obvious harm. However, this requires more research in the future.

Dysbiosis can lead to intestinal inflammation, DNA damage and cell proliferation, increasing the risk of cancer development [34, 35]. In addition, the gut microbiota may also affect the host's metabolic, immune, and signaling pathways, which in turn affects the occurrence and progression of colorectal cancer [36]. Therefore, in-depth study of the relationship between intestinal flora and CRC is very crucial for the prevention and diagnosis of CRC. In our AOM-DSS model of colorectal tumorigenesis, colonization of the flora of NAR-fed group mice significantly increased the abundance of intestinal microbial species. This finding may have important implications for gut health and the treatment of related diseases. To further explore the complex mechanism of how naringenin inhibits CRC progression, we conducted in-depth studies through *in vitro* experiments. Naringenin significantly inhibited the growth of HCT116 and SW480 cell lines in a dose-dependent manner, implying a potential therapeutic window for precise dose control.

Exogenous IL-6 decreased the viability of HCT116 and SW480 cells after naringin treatment. However, IL-1β did not have this effect. Therefore, IL-6 is a crucial mediator of NAR's effect on intestinal cancer cell survival, which deserves further in-depth investigation. Meanwhile, Western blot analysis showed the same trend of p-STAT3 protein expression in the cells. These results suggest that naringenin may inhibit the proliferation of CRC cells by mediating the IL-6/Stat3 signaling pathway, thus providing deeper insights into the underlying mechanisms.

The dysregulation of the IL-6/STAT3 signaling pathway is significantly associated with the progression of numerous human solid tumors. Therefore, extensive exploration is being conducted on the regulation of the IL-6/STAT3 signaling pathway to develop new therapies for CRC [37, 38]. Especially, recent studies have focused on the ability to target the IL-6/STAT3 pathway using small molecule inhibitors or monoclonal antibodies [39, 40]. These therapeutic strategies aim to block the excessive activation of STAT3. By disrupting this signaling axis, it is hypothesized that the growth and progression of CRC can be effectively suppressed. Moreover, preclinical studies have shown promising results, with some inhibitors demonstrating significant anti-tumor activity in CRC models. These emphasize the IL-6/STAT3 pathway as a viable therapeutic target for CRC, and more clinical trials should evaluate the safety of these new treatments.

Fortunately, extensive research is being conducted to discover natural compounds to help prevent and treat this disease. Flavonoids are one of these compounds that have shown promising results. Their potential health benefits have been studied extensively [41]. Studies have provided strong evidence that flavonoids can prevent the growth and spread of cancer cells. Flavonoids have been shown to impact colorectal cancer cells significantly, inhibiting their growth and promoting their death [20]. Flavonoids may inhibit CRC tumor growth by restoring fecal metabolites and protecting intestinal epithelial barrier [42]. Overall, the evidence suggests that flavonoids have significant potential as a natural strategy for preventing and treating colorectal cancer.

The combination of natural medicines with certain therapeutic agents can improve CRC. Typical examples include curcumin [43], resveratrol [44]. In recent years, naringenin has attracted much attention for its anti-cancer function. Especially in human CRC cells, naringenin has shown remarkable anti-cancer properties [20].In *in vivo* experiments, naringenin was observed to significantly inhibit the growth of colorectal tumors by establishing an animal model of colorectal cancer [45-47]. Research has demonstrated that naringenin significantly suppressed tumor growth in a colorectal cancer model, exhibiting minimal toxicity [48]. Ongoing studies are exploring the efficiency of naringenin in combination with other therapeutic agents and its potential to prevent cancer recurrence [49]. A recent study found that naringenin promotes apoptosis in CRC cells by affecting mitochondrial function and activating the AMP-activated protein kinase (AMPK) pathway. Researchers are investigating the optimal dosage and administration methods to maximize its anti-cancer effects while minimizing any potential side effects [50-51].

Naringenin inhibits G0/G1 phase cell proliferation and alters mitochondria-mediated intrinsic apoptotic pathway in triple-negative human breast cancer cells (MDA-MB-231) [18]. The combination of naringenin and 5-fluorouracil reduces the cardiotoxicity and liver injury of 5-fluorouracil in nude mice. The concomitant administration of these two pharmacological agents modifies mitochondrial functionality by elevating the levels of ROS and reducing the MMP, which in turn activates the AMPK/mTOR signaling pathway [50]. Naringenin demonstrated an inhibitory effect on the proliferation and migration of A2780 and ES-2 cancer cell lines *in vitro*, accompanied by a down-regulation of PI3K. Naringin treatment resulted in significant reductions in tumor weight and volume, with oral administration having a more pronounced effect. In addition, naringenin improved the population composition of ovarian cancer animal microbiota [50]. These results further illustrate the generalizability of naringenin’s therapeutic effects in his different cancer types and in different models of colorectal cancer.

*Alistipes* is a potential bacterial marker for CRC diagnosis [52]. However, some link their presence to health promotion, such as the beneficial role of *Alistipes* in colitis and various liver diseases. An important implication is that *Alistipes* promotes CRC through the IL-6/STAT 3 pathway [53].

In our study, it is noteworthy that naringenin treatment increased the abundance of Alistipes. Therefore, animal studies should further explain the mechanisms of its modulation of disease and the role of the genus as a symbiont in various complex multimodal diseases.

Intestinimonas massiliens are recognized for its substantial production of butyrate, a short-chain fatty acid (SCFA) that holds potential medical significance. Butyrate helps maintain homeostasis of colon cells. Additionally, butyrate has been associated with enhancements in insulin sensitivity and glucose homeostasis, similar to the effects observed with other SCFAs [54]. *Parabacteroides* constitutes a fundamental component of the human gut microbiota, exhibiting an average prevalence of 1.27% across twelve distinct human populations. Notable correlations have been documented between *Parabacteroides* and host health [55]. *Roseburia intestinalis* is a probiotic that inhibits intestinal inflammation, prevents colorectal tumorigenesis, and also enhances anti-pd-1 efficacy [56]. Fortunately, in our study, naringenin increased their proportion in the gut microbiota.

This finding provides insight into the pathogenesis of colorectal cancer and points to potential strategies for preventing this disease by modifying diet and gut health. Overall, a multi-faceted approach that combines dietary modifications, probiotic supplementation, and targeted medical interventions holds great promise in the fight against HFD-CRC.

The toxicity of the formulation was assessed by rat hippocampal sections and *in vivo* models of cryptobacteria and zebrafish. Evaluation of analytical parameters such as linearity, limit of detection, limit of quantification, specificity, precision, accuracy and robustness showed that the nanocapsules showed no signs of toxicity [57, 58]. A research investigation was conducted to assess the existing data regarding the impact of flavonoids on oral cancer, with the objective of elucidating the molecular mechanisms that may contribute to their potential anticancer effects [59].

However, there are many shortcomings and limitations in this study. Although naringenin has shown anticancer effects in many experiments, its specific mechanism of action is not clear. The anti-inflammatory effects of naringenin were mainly found in animal experiments and some cellular models, and evidence from clinical trials is lacking. And the small sample size of 8 animals per group limits the statistical power of the study, which may affect the reliability and generalizability of the findings. Subsequently, we will conduct experiments on naringenin in other cancer species with larger sample sizes where feasible to ensure statistical robustness and enhance the reliability of the conclusions. There is still very limited information about the clinical use of flavonoids and naringenin. We look forward to the clinical application of naringenin.

In conclusion, our study demonstrates that NAR treatment affects the composition of the gut microbiota, inhibits the IL-6/STAT3 signaling pathway, and slows the progression of HFD-CRC. FMT confirmed the inhibitory effect of naringenin on colorectal tumor development. This finding establishes a direct relationship between naringenin-regulated gut microbiota and colorectal tumorigenesis associated with a high-fat diet.

## Supplemental Materials

Supplementary data for this paper are available on-line only at http://jmb.or.kr.
