# Supplementary material for: Naringenin Inhibits Colorectal Cancer associated with a High-Fat Diet through Modulation of Gut Microbiota and IL-6/STAT3 Pathway
Source: J Microbiol Biotechnol. 2025 Apr 23;35:e2412029. doi: 10.4014/jmb.2412.12029 (PMC12089958; doi:10.4014/jmb.2412.12029)
Supplement: Supplementary file 1 [file jmb-35-e2412029-supple.pdf]

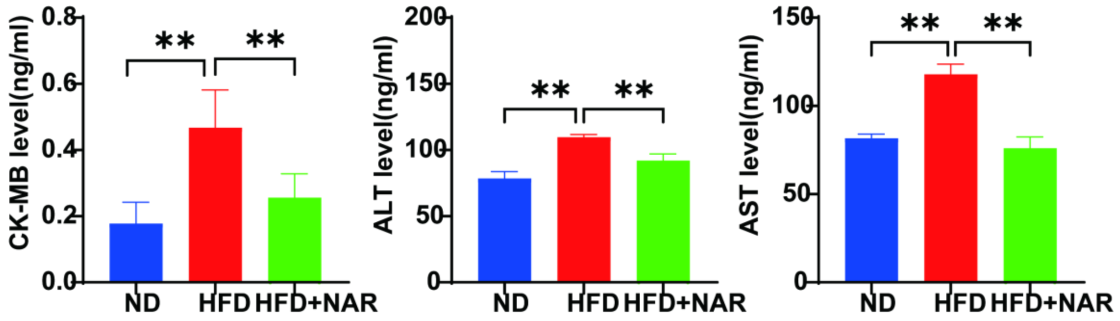

Figure S1:(A) Levels of CK-MB in serum;(B) Levels of ALT in serum;(C) Levels of AST in serum.

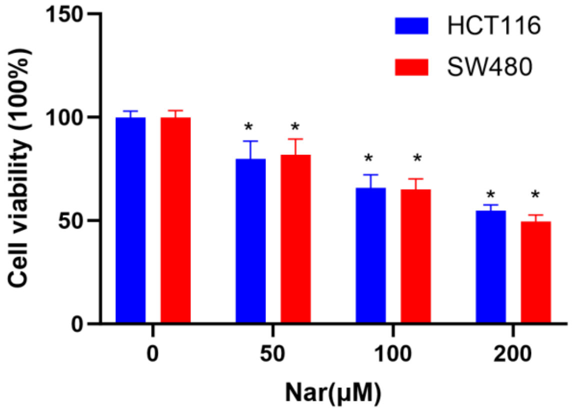

Figure S2: HCT 116 and SW 480 cells are treated with indicated concentrations of NAR and exogenous IL1β for 24 h. Cell viability is measured using the MTT assay system;

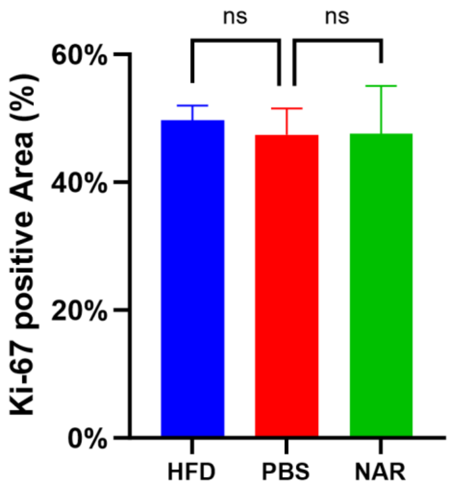

FigureS3: IHC staining of Ki-67 expression.

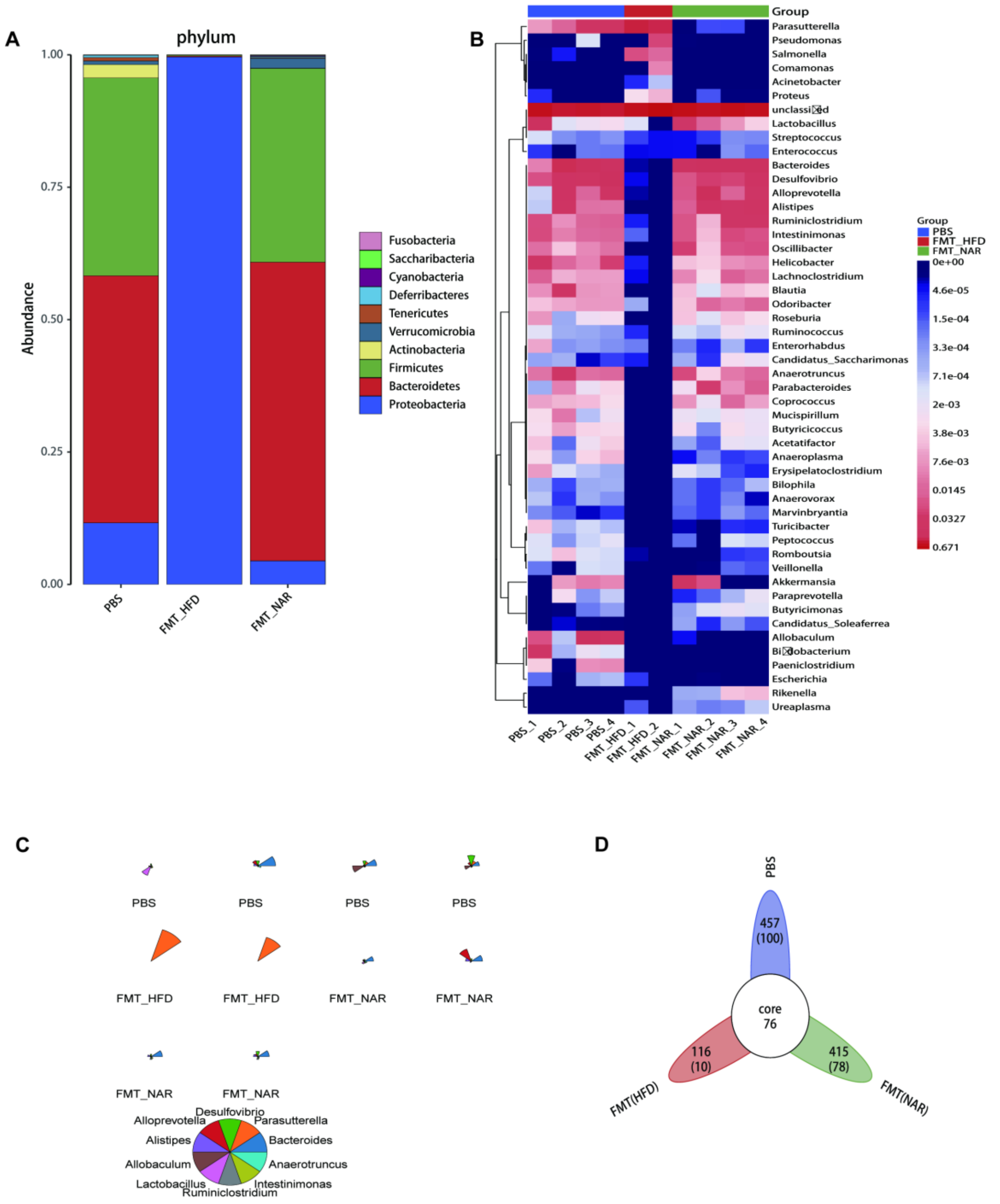

FigureS4:(A) Bacterial taxonomic profiling at the phylum level among samples;(B) Heat map of the relative abundance of the first 30 enriched bacterial OTUs between the three groups;(C) Horizontal star map of the top 10 genera in abundance;(D)Flower plot.
